# Relationship Between Childhood Left-Behind Experience and Quality of Life Among Chinese University Freshmen: Place of Origin Matters

**DOI:** 10.3389/fpsyt.2021.789622

**Published:** 2021-11-24

**Authors:** Hai-Mei Li, Yan-Min Xu, Bao-Liang Zhong

**Affiliations:** Affiliated Wuhan Mental Health Center, Tongji Medical College of Huazhong University of Science and Technology, Wuhan, China

**Keywords:** freshmen, University, left-behind experience, quality of life, place of origin, China

## Abstract

**Background:** Childhood left-behind experience (LBE) has a long-term detrimental effect on the mental health of Chinese University students, but it remains unclear whether childhood LBE negatively impacts the quality of life (QOL) of University students and whether the LBE–QOL association differs between students of rural origin and students of urban origin. This study examined the LBE–QOL relationship and the interactive effect between LBE and place of origin on QOL among Chinese University freshmen.

**Methods:** By using a two-stage random cluster sampling approach, a total of 5,033 freshmen were recruited from two comprehensive universities. The students completed an online, self-administered questionnaire that included sociodemographic variables, a 2-week physical morbidity assessment, and assessments of depressive symptoms, academic stress, and QOL. The Chinese six-item QOL scale was used to assess QOL. Multiple linear regression was used to test the independent LBE–QOL association and the interaction between LBE and place of origin.

**Results:** Students with childhood LBE had significantly lower QOL scores than those without LBE (60.1 ± 13.1 vs. 64.3 ± 11.7, *p* < 0.001). After adjusting for the potential confounding effects of other sociodemographic variables, 2-week physical morbidity, depressive symptoms, and academic stress, childhood LBE was significantly associated with a lower QOL score (β: −3.022, *p* < 0.001) and the LBE–place of origin interaction was still significantly associated with the QOL score (β: −2.413, *p* < 0.001). Overall, compared to non-LBE, LBE was associated with a QOL score decrease of 5.93 among freshmen of urban origin and of 3.01 among freshmen of rural origin.

**Conclusion:** In Chinese University freshmen, childhood LBE is independently associated with poor QOL, and the LBE–QOL association is greater among freshmen from urban backgrounds than among freshmen from rural backgrounds.

## Introduction

In China, the past four decades have witnessed the unprecedented migration of young laborers from impoverished rural villages to thriving coastal cities and concentrated industrial zones, seeking greater employment opportunities, and pursuing a better life for themselves and their families (1). In 2019, the total number of migrant workers reached 290.8 million, or 20.6% of the whole Chinese population (2). Because of the high cost of living, unstable employment, and difficulties in arranging child care and schooling in cities, many migrant parents have to leave their children behind, in the care of grandparents, other relatives, or friends who remain in their rural villages of origin (3). Despite the lack of official, up-to-date statistics, left-behind children have become a large segment of the population in China; for example, according to the All China Women's Federation, there were 61.0 million left-behind children in rural China in 2013 (4).

Early separation from parents, from either one or both parents, has a profound and long-lasting negative impact on both the physical and mental health of children. Accumulating evidence has shown a higher risk of wasting, stunning, slow physical development, poor nutritional status, obesity, depressive symptoms, anxiety symptoms, suicidal ideation, substance use, and poor health-related quality of life (QOL) among left-behind children than among children from intact families (5–10). Importantly, the negative mental health effect of childhood left-behind experience (LBE) does not diminish over time but persists to late adolescence and young adulthood; for example, compared to University students without childhood LBE, those with LBE are at a significantly higher risk for developing depressive symptoms, anxiety symptoms, low self-esteem, suicidal and self-harm behaviors, and other mental health problems (11–15).

It is worth noting that being left behind is not a unique experience for rural Chinese children. In recent years, due to the rapid economic growth, increasing urban-to-urban migration, parental return to universities to pursue advanced degrees, and long-term parental business travel, there has been a substantial increase in the number of urban left-behind children in China (16) as well, 3.1 million in 2000 and 28.3 million in 2015 (17). Empirical studies have revealed that urban left-behind children have significantly more mental health and substance abuse problems than both rural left-behind children and urban non-left-behind children (18, 19), suggesting a potentially high risk of mental health problems in this emerging vulnerable population.

University students, in particular first-year students, are facing the transition period from late adolescence to young adulthood along with the difficulties of adjusting to college life, changing social identities, and forming new social relationships (20). Numerous studies have reported the high prevalence of mental health problems and poor self-rated health among Chinese University students (20–25). In addition to LBE, place of origin is one of the commonly reported factors associated with both physical and mental health in Chinese University students; for example, compared to University students of urban origin, students of rural origin have more mental health problems and poorer mental and physical QOL (26–29). Accordingly, among University students with childhood LBE, it is reasonable to hypothesize that students from rural origins have poorer physical and mental health than students from urban origins. However, findings from two comparative studies do not support this hypothesis. One study reported a similar prevalence of psychological symptoms among rural and urban LBE University students, and the other study reported significantly better mental health among rural LBE students than among urban LBE students (12, 13). This suggests that the effect of LBE on the mental health may differ between University students of rural origin and urban origin. In other words, place of origin may moderate the detrimental effects of childhood LBE on the mental health of University students. Nevertheless, nearly all existing studies on the long-term impact of LBE on the health of University students either did not consider or ignored the differences in health effects of LBE between students of rural origin and those of urban origin (11, 15, 30, 31).

University is an important life phase, when the introduction of targeted health-related interventions has the potential to positively impact both short- and long-term health status and health-related QOL outcomes (32). By definition, QOL is broader than health, and refers to a sense of well-being that encompasses physical health, role functioning, social functioning, and mental health (33). To facilitate the campus-based health policymaking and planning, it is necessary to identify factors associated with QOL and contextual factors that may influence the factor–QOL associations in University students. The present study investigated the relationship between childhood LBE and QOL among Chinese University freshmen and examined whether the relationship varied between students of rural origin and students of urban origin. Based on the above literature review, we speculated that childhood LBE was significantly associated with poorer QOL and that there was a significant interactive effect between LBE and place of origin.

## Methods

### Participants

This study was a cross-sectional survey, which was carried out to investigate QOL and mental health help-seeking behaviors among freshman students at two comprehensive universities, one in Fuzhou and the other in Wuhan, China, between November and December 2019. First-year students who were admitted in the fall of 2019 and of Chinese nationality were invited to join this study. Students who were repeating the academic year and international students were excluded. Participants were selected by using a two-stage random cluster sampling approach. By using a random number table, 22 schools were selected from a total of 42 schools at the two universities. These selected schools had 5,469 first-year students, and all the students were invited to participate in this study.

The survey protocol was approved by the Ethics Committee of Wuhan Mental Health Center. Participants electronically signed the informed consent form first and then were automatically directed to the online survey page.

### Assessments and Procedures

We used a self-administered questionnaire to collect data, and the questionnaire was distributed online *via* the “Questionnaire Star,” a popular platform providing free online survey services in China.

Sociodemographic variables included study site, sex, age, status as an only child in the family, ethnic group, academic major (science vs. liberal arts) (34), marital status of parents, LBE, and place of origin. Students with childhood LBE were those who lived in their original domicile but who did not live together with their parents for a minimum of 6 months before being admitted to the universities because either one parent or both parents migrated elsewhere for work (14, 17). Place of origin referred to the students' household registration location as either a rural or an urban area (17).

We assessed the physical health of respondents by using the 2-week physical morbidity question from China's Multi-wave National Health Services Surveys (35), which asked their experiences of any physical health problems during the past 2 weeks, including infectious diseases and chronic non-communicable diseases.

The validated Chinese 9-item Patient Health Questionnaire (PHQ-9) was used to assess depressive symptoms over the past 2 weeks (36). All items of the PHQ-9 were answered on a four-point scale, from “0 = not at all” to “3 = nearly every day.” The total scores on the PHQ-9 ranged between 0 and 27, with seven or higher denoting clinically significant depressive symptoms (37).

The level of perceived academic stress was assessed with a single question developed by the authors: “What is your level of academic stress?” (high, not high).

The validated Chinese six-item QOL scale was used to assess the QOL of students (33). The scale was developed by Phillips et al. and has been widely used to evaluate QOL in a variety of Chinese populations, including students (33, 38, 39). This scale assesses QOL in terms of six domains: physical health, psychological health, economic circumstances, study, family relationship, and relationship with non-family associates. Each item is rated on a five-point scale, from “1 = very poor” to “5 = very good.” The crude total QOL score ranges from 6 to 30, with a higher score denoting better QOL. As recommended by Phillips et al. (38), the crude total QOL score was further rescaled on a “0–100” scale to obtain the total QOL score.

### Statistical Analysis

The independent-samples *t*-test was used to compare QOL scores between groups according to sociodemographic characteristics. To examine the LBE–QOL association, a multiple linear regression analysis was performed that entered LBE as the main predictor; place of origin, other sociodemographic variables, 2-week physical morbidity, depressive symptoms, and academic stress as the covariates; and QOL score as the outcome variable (“main effect model”). To test whether the LBE–QOL association differed between places of origin, an interaction term, the production of LBE and place of origin, was included as an additional independent variable in the above linear regression model (“interactive effect model”). Statistically significant regression coefficients of LBE in the main effect model and the interaction term in the interactive effect model suggested the presence of the impact of LBE on QOL and the moderating effect of place of origin on the LBE–QOL association. Finally, a graph was used to visualize the interactive effect of LBE and place of origin, where predicted QOL scores by LBE and place of origin from the interactive effect model are shown. Prior to the formal analysis, the assumption of the absence of multi-collinearity was tested. The results of collinearity diagnostics analysis showed that variance inflation factor values of all independent variables ranged between 1.001 and 1.149, much lower than the critical threshold of 10 (40); therefore, no significant multi-collinearity was present among the independent variables in our regression analysis. We performed all statistical analyses using SPSS 25.0, assuming a two-sided test at the 0.05 level of significance.

## Results

A total of 5,033 students completed the survey, for a response rate of 93.0%. The average age of the study sample was 18.5 years [standard deviation (SD): 0.9; range: 15–30 years]; 98.2% were 17–20 years, 54.1% were girls, 29.2% had childhood LBE, and 60.2% were from rural areas. The detailed sociodemographic characteristics, the 2-week physical morbidity, depressive symptoms, and academic stress of freshmen are shown in Table 1.

**Table 1:** Characteristics of freshmen and quality of life (QOL) scores of different groups.

**Characteristics**		***n* (%)**	**QOL score**	***t***	***p***
Site	Wuhan	2,193 (43.6)	63.26 ± 12.14		
	Fuzhou	2,840 (56.4)	62.95 ± 12.41	0.891	0.373
Sex	Male	2,311 (45.9)	63.01 ± 13.80		
	Female	2,722 (54.1)	63.15 ± 10.85	0.415	0.678
Age (years)	<18	260 (5.2)	62.96 ± 11.95		
	≥18	4,773 (94.8)	63.09 ± 12.31	0.163	0.870
The only child	Yes	1,708 (33.9)	64.08 ± 12.93		
	No	3,325 (66.1)	62.58 ± 11.93	3.995	<0.001
Ethnic group	Han	4,698 (93.3)	63.17 ± 12.37		
	Minorities	335 (6.7)	61.92 ± 11.07	1.804	0.071
Academic major	Liberal arts	1,604 (31.9)	62.46 ± 12.76		
	Science	3,429 (68.1)	63.38 ± 12.06	2.479	0.013
Marital status of parents	Married	4,019 (79.9)	63.49 ± 12.26		
	Others^*^	1,014 (20.1)	61.47 ± 12.32	4.686	<0.001
Childhood left-behind experience	Yes	1,471 (29.2)	60.13 ± 13.08		
	No	3,562 (70.8)	64.31 ± 11.74	10.611	<0.001
Place of origin	Urban	2,004 (39.8)	64.22 ± 13.19		
	Rural	3,029 (60.2)	62.33 ± 11.61	5.217	<0.001
Two-week morbidity	Yes	1,294 (25.7)	58.84 ± 11.47		
	No	3,739 (74.3)	64.55 ± 12.23	15.175	<0.001
Depressive symptoms	Yes	1,497 (29.7)	57.04 ± 12.25		
	No	3,536 (70.3)	65.65 ± 11.39	23.266	<0.001
Academic stress	High	1,417 (28.2)	59.22 ± 13.31		
	Not high	3,616 (60.8)	64.60 ± 11.53	13.364	<0.001

* *Others included never-married, remarried, cohabiting, separated, divorced, and widowed*.

The mean QOL score was 63.1 (SD: 12.3, range: 0–100). As displayed in Table 1, students with childhood LBE had significantly lower QOL scores than those without LBE (60.1 ± 13.1 vs. 64.3 ± 11.7, *p* < 0.001), and rural students had significantly lower QOL scores than urban students (62.3 ± 11.6 vs. 64.2 ± 13.2, *p* < 0.001).

In the main effect model (Table 2), after adjusting for the confounding effects of sociodemographic variables, physical health, depressive symptoms, and academic stress, childhood LBE was still significantly associated with a lower QOL score [coefficient (β): −3.022, *p* < 0.001].

**Table 2:** Multiple linear regression on relationship between childhood left-behind experience (LBE) and quality of life (QOL) among freshmen, adjusting for the confounding effects of sociodemographic variables, physical health, depressive symptoms, and academic stress.

**Characteristics**		**Unstandardized coefficient**	**Standard error**	***t***	***p***
Childhood LBE	Yes vs. no	−3.022	0.356	8.490	<0.001
Place of origin	Rural vs. urban	−1.362	0.344	−3.958	<0.001
Site	Fuzhou vs. Wuhan	−0.306	0.320	−0.955	0.340
Sex	Female vs. male	0.410	0.341	1.202	0.229
Age (years)	≥18 vs. <18	−0.464	0.719	−0.645	0.519
The only child	No vs. yes	−1.074	0.359	−2.992	0.003
Ethnic group	Minorities vs. Han	0.059	0.640	0.093	0.926
Academic major	Science vs. liberal arts	0.822	0.360	2.287	0.022
Marital status of parents	Others^*^ vs. married	−1.295	0.398	−3.254	0.001
Two-week morbidity	No vs. yes	−3.607	0.375	−9.625	<0.001
Depressive symptoms	No vs. yes	−7.031	0.361	−19.492	<0.001
Academic stress	Not high vs. high	−3.679	0.360	−10.214	<0.001

* *Others included never-married, remarried, cohabiting, separated, divorced, and widowed*.

**Figure 1:**
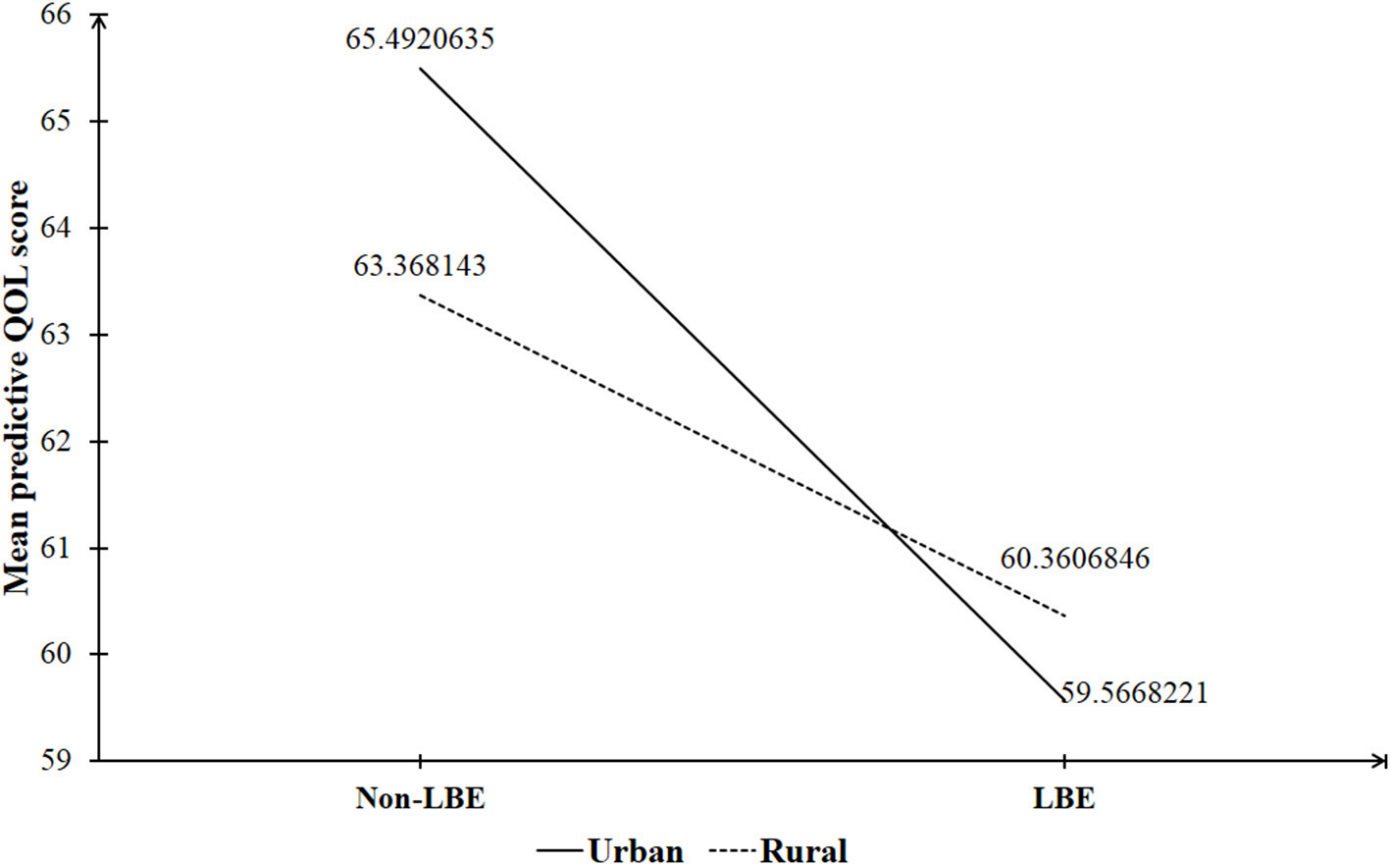
The interactive effect of childhood left-behind experience (LBE) and place of origin on quality of life (QOL) of Chinese University freshmen.

In the interactive effect model (Table 3), after adjusting for the confounding effects of sociodemographic variables, physical health, depressive symptoms, and academic stress, the interactive effect of childhood LBE and place of origin was still significantly associated with the QOL score (β: −2.413, *p* < 0.001). As shown in Figure 1, a one-unit increase in the LBE status, from non-LBE to LBE, was associated with a QOL score decrease of 5.93 among urban freshmen and 3.01 among rural freshmen.

**Table 3:** Multiple linear regression on interactive effect of childhood left-behind experience (LBE) and place of origin on quality of life (QOL) among freshmen, adjusting for the confounding effects of sociodemographic variables, physical health, depressive symptoms, and academic stress.

**Characteristics**		**Unstandardized coefficient**	**Standard error**	***t***	***p***
Childhood LBE × place of origin		−2.413	0.750	−3.218	0.001
Childhood LBE	Yes vs. No	−4.644	0.618	7.529	<0.001
Place of origin	Rural vs. Urban	0.412	0.651	0.638	0.522
Site	Fuzhou vs. Wuhan	−0.308	0.320	−0.964	0.335
Sex	Female vs. male	0.397	0.341	1.165	0.244
Age (years)	≥18 vs. <18	−0.418	0.719	−0.581	0.561
The only child	No vs. yes	−1.012	0.359	−2.818	0.005
Ethnic group	Minorities vs. Han	0.049	0.639	0.077	0.939
Academic major	Science vs. liberal arts	0.833	0.359	2.319	0.020
Marital status of parents	Others^*^ vs. married	−1.287	0.398	−3.238	0.001
Two-week morbidity	Yes vs. no	−3.588	0.374	−9.582	<0.001
Depressive symptoms	Yes vs. no	−7.026	0.360	−19.495	<0.001
Academic stress	High vs. not high	−3.663	0.360	−10.179	<0.001

* *Others included never-married, remarried, cohabiting, separated, divorced, and widowed*.

## Discussion

To the best of our knowledge, this is the first large-scale study in China that examined both the negative impact of childhood LBE on QOL among University students and the interactive effect of childhood LBE and place of origin on the QOL of freshmen. In the Chinese general population, the normative QOL score, as measured by the Chinese six-item QOL scale, was 70.8 (41). Compared to the normative data, we found a significantly and substantially lower QOL score in University freshmen than in the general population (63.1 vs. 70.8, *t* = −44.705, *p* < 0.001), suggesting the poor QOL of Chinese University freshmen and the urgent need to develop programs to improve QOL in this population.

As shown in Table 1, many factors other than LBE were also associated with QOL among freshmen; therefore, it is important to consider the possible confounding effects of these factors on the LBE–QOL association. In the main effect model, the significant LBE–QOL association suggests that LBE was independently associated with poor QOL among University freshmen. In previous University students-based studies, childhood LBE was reported to be significantly associated with mental health problems (11–15), but in the present study, we revealed the independent LBE–QOL association among University freshman students, extending the long-term health outcomes of childhood LBE from mental health problems to worsened QOL.

Parents' company and supervision play a vital role in the development and functioning of their children and in preparing them to manage the challenges they will confront in their academic, social, occupational, and cultural lives as adults (42). According to the attachment theory (43), the early absence of parents disrupts the development of secure attachment bonds between children and their parents, which has a long-term negative effects on a child's psychological development in terms of personality, cognition, psychopathology, resilience, and coping style. Therefore, children with LBE are more likely to experience mental health problems, maladaptive behaviors, adjustment problems, poor coping skills, and interpersonal difficulties after they enter the universities. These findings may explain the association of childhood LBE with poor QOL among University freshmen.

The greater LBE–QOL association among urban freshmen than among rural freshmen observed in this study is similar to the greater negative effect of childhood LBE on mental health among urban freshmen than among rural college freshmen observed in a prior study (13). Because the interactive effect of LBE and place of origin was independent of sociodemographic variables, physical health, depressive symptoms, and academic stress, we speculated that some place-specific contextual factors may magnify or mitigate the detrimental effect of childhood LBE on QOL among University freshmen. First, in China, the urban community is a “stranger” society, but the rural community is an “acquaintance” or “relationship” society where the cultural value is still deeply influenced by Confucianism and the patriarchal clan system (44). Despite a lack of or insufficient parental care and company, urban left-behind children are less likely than rural left-behind children to obtain support and supervisions from relatives. Second, compared to rural children, urban children are more likely to be exposed to the internet, new types of drugs, and electronic games; therefore, urban left-behind children are at greater risk than rural left-behind children for developing addictive behaviors. The effects of weaker care from relatives and a higher prevalence of addictive behaviors among urban left-behind children than among rural left-behind children may persist into young adulthood and result in the poorer QOL reported among urban than rural LBE University freshmen. Third, rural left-behind children who pass the National College Entrance Examination and become University students represent the most successful fraction of this vulnerable population. Because of difficulties experienced during childhood, these rural LBE students are more resilient, capable of living independently, and adaptive to new environments than urban LBE students. These straits may partly offset the negative effect of childhood LBE, possibly leading to the relative health disadvantages observed among urban but not among rural LBE students.

This study has two limitations. First, characteristics of childhood LBE were not assessed in detail. Since the relationship with the caregiver during the left-behind period, the age at onset of being left behind, and the length of the left-behind period are associated with depressive symptoms in Chinese LBE University students (45), it remains unclear whether the poorer QOL among urban LBE students was related to their characteristics of LBE or other factors. Second, childhood LBE was retrospectively assessed in this study; therefore, recall bias in the measurement of LBE may exist.

In summary, in Chinese University freshmen, childhood LBE is significantly associated with poor QOL, and the LBE–QOL association is greater among freshmen of urban origin than among freshmen of rural origin. Freshmen with LBE, in particular freshmen of urban origin with LBE, could be considered as a target group of campus-based QOL promotion programs. Campus-based programs designed to improve QOL among Chinese University freshmen may include health education, psychosocial support, social skills training, and stress-management training.

## Data Availability Statement

The raw data supporting the conclusions of this article will be made available by the authors, without undue reservation.

## Ethics Statement

The studies involving human participants were reviewed and approved by Ethics Committee of Wuhan Mental Health Center. The patients/participants provided their written informed consent to participate in this study.

## Author Contributions

H-ML: acquisition and analysis of data for the study, drafting the paper, and interpretation of data for the study. Y-MX: design and acquisition of data for the study. B-LZ: drafting the paper, revising the paper for important intellectual content, and interpretation of data for the study. All authors contributed to the article and approved the submitted version.

## Funding

This work was supported by the National Natural Science Foundation of China (Grant Number: 71774060). The funding source listed had no role in study design, in the collection, analysis, and interpretation of data, in the writing of the report; and in the decision to submit the paper for publication.

## Conflict of Interest

The authors declare that the research was conducted in the absence of any commercial or financial relationships that could be construed as a potential conflict of interest.

## Publisher's Note

All claims expressed in this article are solely those of the authors and do not necessarily represent those of their affiliated organizations, or those of the publisher, the editors and the reviewers. Any product that may be evaluated in this article, or claim that may be made by its manufacturer, is not guaranteed or endorsed by the publisher.
